# Biologic Roles of Estrogen Receptor-***β*** and Insulin-Like Growth Factor-2 in Triple-Negative Breast Cancer

**DOI:** 10.1155/2015/925703

**Published:** 2015-03-22

**Authors:** Nalo Hamilton, Diana Márquez-Garbán, Vei Mah, Gowry Fernando, Yahya Elshimali, Hermes Garbán, David Elashoff, Jaydutt Vadgama, Lee Goodglick, Richard Pietras

**Affiliations:** ^1^UCLA School of Nursing, Los Angeles, CA 90095, USA; ^2^UCLA Jonsson Comprehensive Cancer Center, Los Angeles, CA 90095, USA; ^3^Division of Hematology-Oncology, Department of Medicine, UCLA David Geffen School of Medicine, Los Angeles, CA 90095, USA; ^4^Department of Pathology and Laboratory Medicine, UCLA David Geffen School of Medicine, Los Angeles, CA 90095, USA; ^5^Division of Cancer Research and Training, Department of Medicine, Charles Drew University School of Medicine and Science, Los Angeles, CA 90095, USA; ^6^Division of Dermatology, Department of Medicine, UCLA David Geffen School of Medicine, Los Angeles, CA 90095, USA; ^7^Division of General Internal Medicine, Department of Medicine, UCLA David Geffen School of Medicine, Los Angeles, CA 90095, USA

## Abstract

Triple-negative breast cancer (TNBC) occurs in 10–15% of patients yet accounts for almost half of all breast cancer deaths. TNBCs lack expression of estrogen and progesterone receptors and HER-2 overexpression and cannot be treated with current targeted therapies. TNBCs often occur in African American and younger women. Although initially responsive to some chemotherapies, TNBCs tend to relapse and metastasize. Thus, it is critical to find new therapeutic targets. A second ER gene product, termed ER*β*, in the absence of ER*α* may be such a target. Using human TNBC specimens with known clinical outcomes to assess ER*β* expression, we find that ER*β*1 associates with significantly worse 5-year overall survival. Further, a panel of TNBC cell lines exhibit significant levels of ER*β* protein. To assess ER*β* effects on proliferation, ER*β* expression in TNBC cells was silenced using shRNA, resulting in a significant reduction in TNBC proliferation. ER*β*-specific antagonists similarly suppressed TNBC growth. Growth-stimulating effects of ER*β* may be due in part to downstream actions that promote VEGF, amphiregulin, and Wnt-10b secretion, other factors associated with tumor promotion. *In vivo*, insulin-like growth factor-2 (IGF-2), along with ER*β*1, is significantly expressed in TNBC and stimulates high ER*β* mRNA in TNBC cells. This work may help elucidate the interplay of metabolic and growth factors in TNBC.

## 1. Introduction

Breast cancer (BC) is the most common malignancy in women [1, 2]. About 70% of patients with breast cancer express estrogen receptor-*α* (ER*α*). Due to the success of endocrine therapies, the mortality of patients with ER*α*-positive tumors has declined significantly in the past decade. Similarly, about 15% of patients have tumors that overexpress HER2 receptor and thus are candidates for HER2-targeted treatments. In contrast, TNBC occurs in 10–15% of patients, yet this disease subtype accounts for about half of all breast cancer deaths. TNBCs lack clinical expression of ER*α*, progesterone receptor, and HER2 overexpression (ER*α*−/PR−/HER2−). TNBCs have incomplete overlap with basal-like breast tumors, a subgroup of breast cancers defined by gene-expression profiling that express specific cytokeratins, and with some hereditary breast cancers. Though heterogeneous, TNBCs typically occur in younger women and African American women as well as among some patients with BRCA1 gene defects [1, 2]. Population-based data show that African American women have a higher incidence of TNBC and present with more advanced stages than Caucasian women [3]. This cancer subtype also associates with adverse biological features including high mitotic count and very aggressive behavior. Of note, some recent reports indicate that the incidence of ER*α*-negative BC and TNBC, high-risk breast cancer subtypes, may correlate with the extent of African ancestry [4]. Though initially responsive to chemotherapy, TNBCs tend to relapse and metastasize early and have a prognosis worse than other subtypes. Currently specific therapies for TNBC are unavailable [1–3].

Estrogens promote progression of ER*α*-positive cancers, effects exerted by binding of estradiol to ER*α*, a ligand-activated transcription factor [5]. ER*α* is predominantly a nuclear-localized protein. Immunohistochemical (IHC) detection of nuclear ER*α* in tumors is a standard clinical assay used to plan patient management [6]. Of special note, recent reports show that a second type of estrogen receptor, termed estrogen receptor-beta (ER*β*), is expressed in TNBC cells [7, 8]. ER*α* and ER*β* are encoded by two different genes, yet ER*β* has 96% homology with ER*α* at the DNA-binding domain and 60% homology at the ligand-binding domain (LBD). However, it is important to note that ER*β* is not identified in standard assays for ER*α*. The role of ER*β* in breast cancer remains to be elucidated but some studies show ER*β* is a biomarker related to a more aggressive clinical course [8] and correlates with Ki-67, a marker of proliferation [7, 9]. Early studies demonstrate higher levels of ER*β* in breast tumors of African American as compared to Caucasian women, suggesting that ER*β* may play a critical role in TNBC development [10–14].

Based on current data, estradiol regulates gene expression by genomic and nongenomic inputs [15, 16]. Genomic signals involve direct action of nuclear-localized ER*α* as an estradiol-regulated transcription factor or coregulator. By contrast, nongenomic signaling involves extranuclear events mediated by extranuclear ERs often in cooperation with coactivator or adaptor proteins [17]; these then impact gene expression indirectly by modulating signaling cascades such as MAPK, PI3K/AKT, and mTOR [8, 14–19] to regulate transcription [5, 15, 16]. In target cells, extranuclear ER*α* forms are derived from the same transcript as nuclear ER*α*; however minor extranuclear ER*α* splice variants occur [15, 16]. In TNBC, less is known about the role of ER*β* in cancer progression [7, 8, 11]. Several ER*β* isoforms occur in breast cancers, including ER*β*1, ER*β*2, ER*β*4, and ER*β*5, but only ER*β*1 retains an intact LBD to interact with specific ligands, thus ER*β*1 is a preferred clinical target [17, 20–22]. ER*β* forms occur in tumor cell nuclei but, as ER*α* forms, may also occur at extranuclear sites [15, 16, 23]. Like ER*α*, ER*β* activates transcription by genomic pathways or nongenomic pathways by interaction with coactivators/coregulators [17] that in turn modulate signaling cascades to impact gene expression and tumor progression [5, 12, 14, 20, 21, 23]. Of note, ER*β* target genes appear to be those that regulate cell death and survival, cell movement, and cell development, growth, and proliferation, as well as genes involved in the Wnt/*β*-catenin and the G1/S cell cycle phase checkpoint pathways [24].

Obesity and the metabolic syndrome are associated with multiple factors that may cause an increased risk for cancer and cancer-related mortality [25]. One example is the insulin family of proteins which have pleiotropic effects on metabolism and growth. A large body of evidence indicates the insulin/insulin-like growth factor (IGF-1, IGF-2) pathway in breast cancer progression [25–29]. Of note, IGF-2 occurs in an unprocessed (pIGF-2) and mature (mIGF-2) form and plays a role in breast cell proliferation and inhibition of apoptosis [28–33]. Under normal conditions, IGF-2 is tightly bound and sequestered [34–38], but overexpression of IGF-2 is associated with breast cancer development and increased tumor formation [39, 40]. Most human cancers overexpress both the IGF-1 receptor (IGF-1R) and insulin receptor (IR) isoforms, leading to the formation of hybrid IGF-1R/IRs. IGF-2 is a known ligand for these receptors as well as the mannose-6-phosphate/IGF-2R and high-affinity binding proteins [25–29, 32]. IGF-2 expression is strongly enhanced in invasive breast cancers and downstream mTOR signaling is stimulated [41] as is TNBC cell migration [29]. Of note, disparities in the expression of IGF-2 and its receptors are reported to occur in breast tissue samples from African American women as compared to Caucasian women and may contribute to differences in clinical outcomes [42]. IGF-2 is detected in both tumor stroma and epithelial breast cancer cells and correlates with both breast epithelial [27, 43–45] and stromal cell proliferation [46]. Westley and May [26] also report that estrogen signaling may cross communicate with IGF pathways, with estrogen promoting increased breast cancer production of IGF-2.

This report details interactions of ER*β* with IGF-2 and other growth factor pathways in TNBC [46–52]. Our findings using TNBC models and archival specimens suggest that IGF-2 may regulate ER*β* expression which in turn modulates metabolic and growth factor pathways in cancer progression.

## 2. Materials and Methods

### 2.1. Breast Cancer Cell Lines

For these studies, we used the following triple-negative breast cancer cell lines (ATCC) which have been previously well characterized as lacking expression of ER*α* and PR as well as overexpression of HER2 [47, 48]: MDA-MB-231, MDA-MB-435, BT549, HCC38, HCC1143, HCC1937, and HCC1806. As controls, we used MCF-7 (expressing abundant ER*α* and minimal/no ER*β*) and T47D (expresses ER*α* and more abundant ER*β*). Cell cultures were routinely maintained at 37°C in a 5% CO_2_ incubator using RPMI 1640 media supplemented with penicillin/streptomycin (10,000 units/mL penicillin and 10,000 units/mL streptomycin sulfate) and 10% fetal bovine serum unless other specific media were recommended by the supplier (ATCC). For the MDA-MB-231 cell line, we created stable transfectants with a specific ER*β* shRNA producing a knockdown of ER*β*. As controls, we used a stable transfectant with a scrambled shRNA and vector control prepared as detailed below (all reagents from Origene).

### 2.2. Reagents

ER*β* ligands for use in these experiments included the following: (a) diarylpropionitrile (DPN), an ER*β* agonist (Tocris), (b) 4-[2-phenyl-5,7-*bis*(trifluoromethyl)pyrazolo[1,5-*a*]pyrimidin-3-yl]phenol (PHTPP), ER*β* antagonist, and (c) 4,4′,4′′-(4-propyl-[1*H*]-pyrazole-1,3,5-triyl)trisphenol (PPT), an ER*α* agonist [49, 50].

### 2.3. Assays for Cell Proliferation

In experiments to assess proliferative effects of ER*β* ligands, cells were grown in phenol red-free, estrogen-free media with 0.1% dextran-coated charcoal-treated- (DCC-) FBS for 48 hours and then treated with selected doses of DPN, PPT, or PHTPP. Cell counts and viability tests (Trypan blue) were done every 24 hours for 3 days. After 72 hours, proliferation was assessed using the BrdU cell proliferation ELISA (Roche). Cell numbers were also assessed initially by cell counts to confirm ELISA data.

### 2.4. Assays for Growth Factor Secretion

Tumor cells were cultured in estrogen-free media and then treated 20–120 minutes with DPN, followed by harvest of particle-free media and application of established ELISA assays for VEGF, amphiregulin, WNT 10b/12 [51–53], signaling molecules that activate angiogenesis, EGFR, and WNT pathways, respectively, which promote TNBC [1, 52, 53].

### 2.5. Knockdown of ER*β* Expression

To suppress ER*β* expression, we used the HuSH 29 mer shRNA constructs (Origene) designed to target human ER*β* (*ESR2*) and included positive and negative controls. Plasmids were designed and validated specifically to knockdown expression of specific genes by RNA interference and allow for enrichment of transfected cells. Each vector expresses a short hairpin RNA (shRNA) under control of the U6 promoter and puromycin resistance gene to select stably transfected cells. Cells were transfected with negative control, scrambled negative control, and shRNA plasmids for ER*β* using MegaTran 1.0 transfection reagent (Origene). After 48 hours, cells were replated at low density in the presence of an effective concentration of puromycin. Culture medium was replaced every 2-3 days, with cells replated every week for 2 weeks. As stable transfectants were obtained, we isolated total RNA to identify colonies with optimal ER*β*1 knockdown, as confirmed by qRT-PCR and immunoassays. Expression of transcripts was done as before [14] and protein levels of ER*β* variants were determined (data not shown). After ER*β*1 knockdown, cell proliferation was determined by established methods in the presence of vehicle or specific ER*β* ligands.

### 2.6. ER*β* Expression by Quantitative RT-PCR (QRT-PCR)

To assess ER*β* transcript levels in TNBC cells [54], total RNA was isolated by the Aurum total RNA mini kit (Bio-Rad). UV spectroscopy and RNA quality indicator (RQI) values obtained from the Experion automated electrophoresis system (Bio-Rad) were used to determine RNA integrity. Primer pairs used for ER*β* (Qiagen SA Biosciences): ER*β* forward: 5′-GCTCATCTTTGCTCCAGATCTTG-3′ and ER*β* reverse: 5′-GATGCTTTGGTTTGGGTGATTG-3. cDNA was synthesized using the iScript cDNA synthesis kit (Bio-Rad) with 400 ng total RNA. The iQ SYBR Green Supermix (Bio-Rad) was used for PCR amplification. Each reaction was performed in triplicate and ribosomal protein 36B4 mRNA, a housekeeping gene unaffected by estrogen, was used as the internal control. The cycling conditions consisted of 95.0°C for 30 seconds followed by 39 cycles of 95.0°C for 5 seconds, 57.0°C for 15 seconds, and 72.0°C for 90 seconds in a CFX96 Touch Thermocycler (Bio-Rad). Transcript levels of ER*β* were normalized to 36B4. Fold induction or repression was measured relative to controls and calculated after adjusting for 36B4 RNA (endogenous control) using 2^−ΔΔCt^, where ΔCt = Ct interested gene − Ct 36B4 RNA and ΔΔCt = ΔCt treatment − ΔCt vehicle control. For experiments that evaluated IGF-2 effects on ER*β*, cell lines were seeded and cultured to 70–75% confluence in complete media followed by 24 hours in serum/phenol red-free media. Following serum starvation, cultures were treated with 100 ng/mL of human recombinant pIGF-2 or mIGF-2 (GroPep) for 24 hours in serum-free, phenol-red-free media before total RNA isolation.

### 2.7. Gel Electrophoresis and Immunoblotting

TNBC cells were maintained in estrogen-free conditions 48 hours before experiments. Cells were then incubated with vehicle control or 10 nM DPN for 15 minutes or 24 hours and then harvested and lysed. Total cell proteins were resolved by 4–15% SDS-PAGE, transferred to polyvinylidene difluoride membranes, and probed with antibodies directed against phosphotyrosine-1068-EGFR (Cell Signaling Technology D7A5), total EGFR (Calbiochem, GR15), or HER-3 (Santa Cruz Biotechnology, C17). Phospho-p44/p42 MAPK (Thr202/Tyr204; #9101), total MAPK (#9102), phospho-mTOR (Ser-2448, clone D9C2), and total mTOR (7C10) antibodies were from Cell Signaling Technology. Proteins were detected by using horseradish peroxidase (HRP) conjugated secondary antibodies and Thermo Scientific Pierce ECL Western Blotting Substrate with enhanced chemiluminescent for detection of activity from HRP. Membranes were stripped and reprobed with *β*-actin as a loading control; anti-beta-actin antibody C4 was from Santa Cruz Biotechnology (sc-47778). Immunoblots shown in figures are a representative of at least three independent experiments.

### 2.8. Patients and Analyses of Archival TNBC Specimens

Overall 19 TNBC cases were available for this study, with 14 provided by tumor banks associated with the UCLA Early Detection Research Network [55, 56] and the Division of Cancer Research at Charles Drew University School of Medicine and Science (CDU) [57]. Patient specimens were obtained from archival breast cancer studies between 1995 and 2007. This study was approved by our Institutional Review Boards and written informed consent was obtained from all participants. TNBC was confirmed by surgical biopsy/pathology and follow-up data. Of the 14 UCLA-CDU cases, each is linked to deidentified clinical and outcome data. Follow-up time was performed up to 5 years. The specimens include those from 11 non-Hispanic Caucasian and 3 non-Hispanic African American female patients. General characteristics of these patients were reported previously [55–57].

An additional 15 specimens were provided by the NCI-supported Cooperative Human Tissue Network (http://www.chtn.nci.nih.gov/). These archival specimens included 5 cases from female patients with TNBC as well as controls for comparison from female patients with ER*α*-negative breast cancer (non-TNBC) (*n* = 5) and ER*α*-positive breast cancer (*n* = 5). Overall, all histologies included cases with invasive tumors with some associated with metastases. Among the 15 NCI CHTN tumor cases, tissue samples were taken from neighboring regions of nonmalignant tissue (*n* = 9). All specimens were collected with the appropriate institutional human subject protection committee approvals and patient consent.

Formalin-fixed and paraffin-embedded tissue samples were stained using standard IHC protocols as before [55–62]. Antibodies used included ER*α* (clone 1D5, DAKO); ER*β*, anti-ER*β* (GeneTex); anti-ER*β*-1 clone PPG5/10 (AbD Serotec); IGF-2 (Abcam); EGFR; and Ki-67 (DAKO). Appropriate controls were included to assess specificity and validate each antibody including (i) appropriate preimmune control, (ii) dose-dependent titration, (iii) known positive and negative tissues, (iv) use of specific peptide competitors, and (v) other established approaches in our laboratory [58, 62]. For IHC, antibody binding was detected by using the “Envision+” System-HRP (DAB) followed by chromogen detection with diaminobenzidine (DAKO). Sections were counterstained with Harris hematoxylin, followed by dehydration through graded alcohol solutions, and mounted. Images were captured by using an Olympus BX41 microscope hooked to a Pixera Pro 150 ES or Evos xl Core microscope. Care was taken to evaluate staining of specific target structures only. Both intensity of staining and percentage of cells were recorded for both neoplastic and normal cells expressing antigen in both nuclear and extranuclear localizations. Board-certified pathologists quantified expression as before [55–58]. We used an Allred scoring system to quantify expression in tumors [62]. Of note, stromal expression of growth factors or steroid receptors in endometrial cancer are reported to be biomarkers to predict response to hormonal therapy [63]. Hence, we explored stromal as well as tumor expression of selected biomarkers, particularly ER*β*1 and IGF-2 (Abcam).

### 2.9. Statistical Analyses

For* in vitro* work, experiments were done in triplicate. Student's *t*-test, ANOVA, or Kruskal-Wallis test, if outcomes were nonnormally distributed, was used to compare intervention groups. Analyses were evaluated using bar and scatter graphs with means, SD, and SE. Time trend curves for agents under different conditions were obtained as appropriate. Repeated measures ANOVA were used to assess time, condition, and time by condition interaction effects. *P* < 0.05 was considered significant. For analyses of clinical TNBC specimens, we used methods and protocols for assessing novel associations with clinical/pathological variables as well as marker associations as detailed before [56–62]. The log-rank test was used to compare overall survival (OS) between those subjects with positive nuclear ER*β*1 expression score versus those with negative scores.

## 3. Results

### 3.1. ER*β* Expression in Archival TNBC Specimens

**Figure 1:**
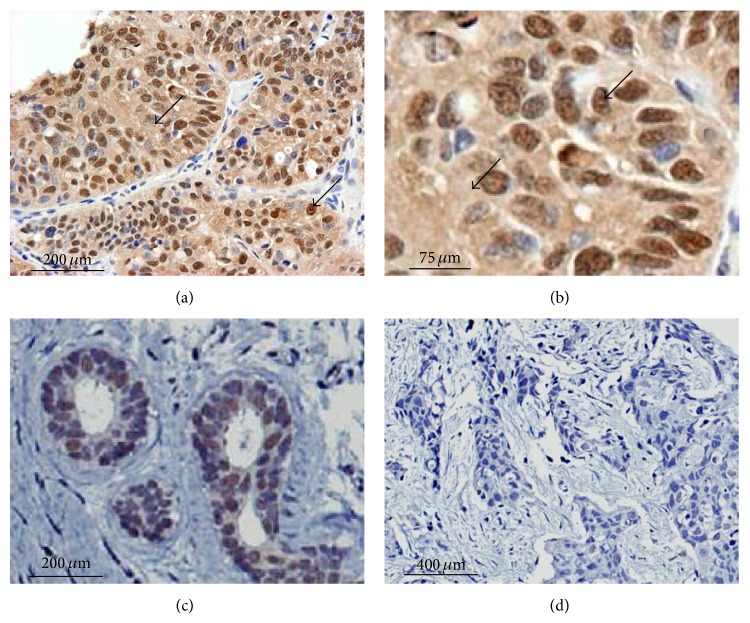
ER*β*1 expression in archival TNBC specimens. Representative examples are shown of IHC staining of tumor and nonmalignant tissue specimens using anti-ER*β*1 antibody (AbDSerotec PPG5/10). (a) TNBC specimen shows nuclear (and cytoplasmic) immunostaining of ER*β*1 at low magnification. (b) The same TNBC specimen shows nuclear (and cytoplasmic) immunostaining of ER*β*1 at higher magnification. (c) Expression of nuclear ER*β*1 is also observed in neighboring nonmalignant mammary tissue from the clinical specimen used in panels (a) and (b). (d) Negative ER*β*1 detected in a different clinical specimen as shown as comparison. Antibody binding was detected by using the “Envision+” amplification system followed by chromogen detection with diaminobenzidine (DAKO). Sections were counterstained with Harris hematoxylin followed by dehydration through graded alcohol solutions and mounting. See Table 2 for a summary of findings on all TNBC cases examined.

**Figure 2:**
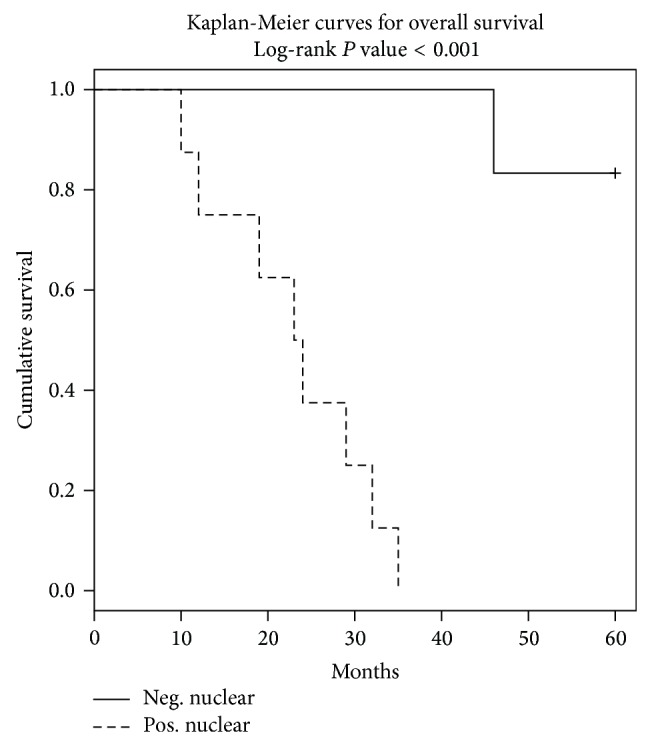
ER*β*1 expression reduces overall survival (OS) in TNBC. TNBCs from 3 African American and 11 Caucasian women were scored for nuclear ER*β*1 using IHC with validated antibody (see Table 2). Allred scores >2 are denoted as positive. In this group of patients with advanced TNBC, overall survival (OS) was significantly worse for TNBC patients with high nuclear ER*β*1 (positive) as compared to those with low (negative) ER*β*1 (*P* < 0.001). We noted that TNBCs from all 3 African American women were ER*β*1-positive.

Several reports indicate that ER*β* expression in node-positive breast cancer is a biomarker for more aggressive disease [8, 21]. For these studies, we used 14 archival TNBC specimens with demographic characteristics including American Joint Committee on Cancer (AJCC) stages provided in Table 1. Using immunohistochemistry, specific ER*β*1 staining was observed and scored in nuclear sites, with our observations noted in Table 2. Representative immune staining patterns are shown in Figure 1. Of note, we also observed evidence of diffuse extranuclear ER*β*1 staining in most specimens but this staining was not scored or analyzed for this report. In further analyses, we assessed the clinical outcome of 14 patients with a 5-year follow-up whose tumors expressed or did not express ER*β*1. In this group of patients with advanced TNBC, overall survival (OS) was significantly worse for TNBC patients with high nuclear ER*β*1 (positive) as compared to those with low (negative) ER*β*1 (*P* < 0.001; see Figure 2). Finally, we note that, of the African American patients included in our sample of 14 TNBC patients (11 Caucasian and 3 African American women), all three had tumors that were ER*β*1-positive.

### 3.2. ER*β* Expression in Breast Cancer Cell Lines

**Figure 3:**
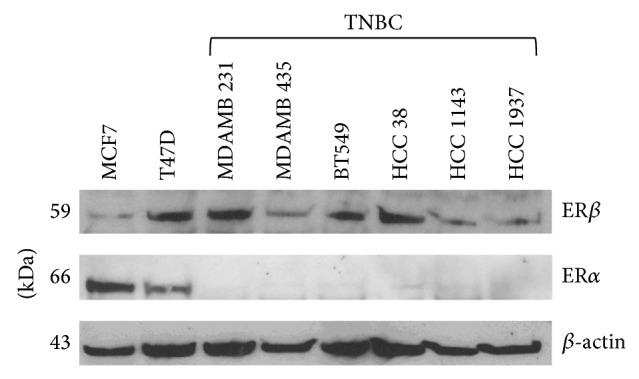
ER*β* is expressed in TNBC cells. Lysates of reported TNBC cells (MDA-MB-231, MDA-MB-435, BT549, HCC38, HCC1143, and HCC1937) and nuclear ER*α*-positive controls (MCF-7, T47D) were subjected to polyacrylamide gel electrophoresis and immunoblots with anti-ER*β* antibody (D7N, Zymed/Invitrogen; confirmed with GeneTex ER*β*1 antibody (not shown)) and anti-ER*α* antibody (1D5, DAKO). T47D cells are also a positive control for ER*β* expression. *β*-actin is used as a loading control. Methods were as before [60, 61]. Blot shown is a representative of at least three independent experiments.

We assessed ER*β* expression in a panel of established TNBC [47, 48] and control breast cancer cell lines, MCF-7 and T47D. All TNBC cell lines were confirmed to express ER*β*. Lysates of reported TNBC cells (MDA-MB-231, MDA-MB-435, BT549, HCC-38, HCC-1143, and HCC-1937) and nuclear ER*α*-positive controls (MCF-7, T47D) were subjected to gel electrophoresis and immunoblots with anti-ER*β* antibody and anti-ER*α* antibody. Results are shown in Figure 3. T47D cells were also used as a positive control for ER*β* expression. These findings indicate significant expression of ER*β* TNBC cell lines.

### 3.3. ER*β* Regulates Breast Cancer Cell Proliferation

**Figure 4:**
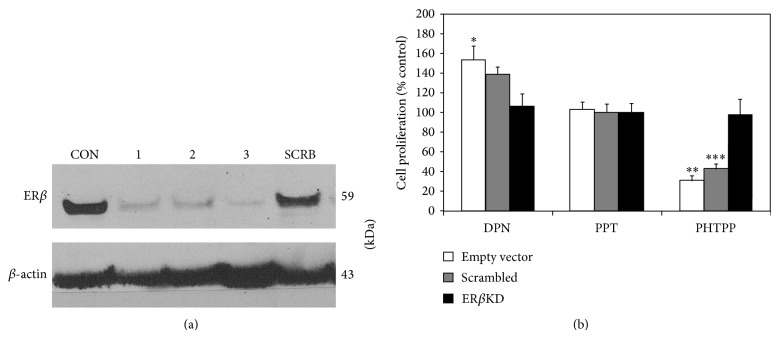
Blockade of ligand-induced proliferation of triple-negative MDA-MB-231 cells by ER*β* antagonists and by ER*β* shRNA. (a) Estrogen receptor-*β* knockdown. MDA-MB-231 TNBC cells were transfected with shRNA control (CON), ER*β*-targeted shRNA (1, 2, 3), and scrambled shRNA vectors (SCRB) (Origene #TG320347). Stable transfectants were selected using puromycin. Cell lysates were processed for Western blot, with results shown for a negative control (CON), 3 different ER*β* knockdown clones (1, 2, and 3), and scrambled shRNA (SCRB). *β*-actin was used as a loading control. (b) Blockade of ligand-induced proliferation of triple-negative MDA-MB-231 cells by ER*β* antagonists and by ER*β* shRNA. Cells were stably transfected with empty vector (white bars), nonspecific shRNA scrambled-sequence plasmid (grey bars), or an ER*β* shRNA plasmid (black bars). As indicated in the figure, cells were treated with different ligands: DPN (ER*β*1 agonist), PPT (ER*α* agonist), and PHTPP (ER*β* antagonist) [49, 50]. Cell proliferation was determined after 72 hours using the BrdU cell proliferation ELISA (Roche) (*n* > 3). Graph shows percentage of surviving cells relative to untreated controls (vehicle control), defined as 100% for each transfection condition: empty vector, scrambled, and ER*β* shRNA. ^*^ *P* = 0.007, ^**^ *P* = 0.005, and ^***^ *P* = 0.006. Data represents at least three independent experiments.

MDA-MB-231 TNBC cells were stably transfected with an empty control vector, nonspecific shRNA scrambled sequence control plasmid, or an ER*β*-specific shRNA plasmid to suppress ER*β* expression. Evidence of expected molecular alterations in ER*β* protein expression in MDA-MB-231 cells is shown in Figure 4(a). Transfected cells were treated with known estrogen receptor agonists and/or antagonists [49, 50] including DPN (ER*β* agonist), PPT (ER*α* agonist), and PHTPP (ER*β* antagonist) alone. As shown in Figure 4(b), PHTPP decreased proliferation in ER*β*-expressing cells but not in cells where ER*β* expression was suppressed. Notably, DPN (ER*β* agonist) increased proliferation only in cells expressing ER*β* (Figure 4(b)). These results are consistent with the hypothesis that ER*β*1 plays a significant role in modulating TNBC proliferation. This finding is consistent with reports that associate ER*β*1 expression with Ki-67, a marker of cell proliferation, in ER*α*-null tumors [17].

### 3.4. ER*β*1 Stimulates Secretion of Growth Factors in TNBC

**Figure 5:**
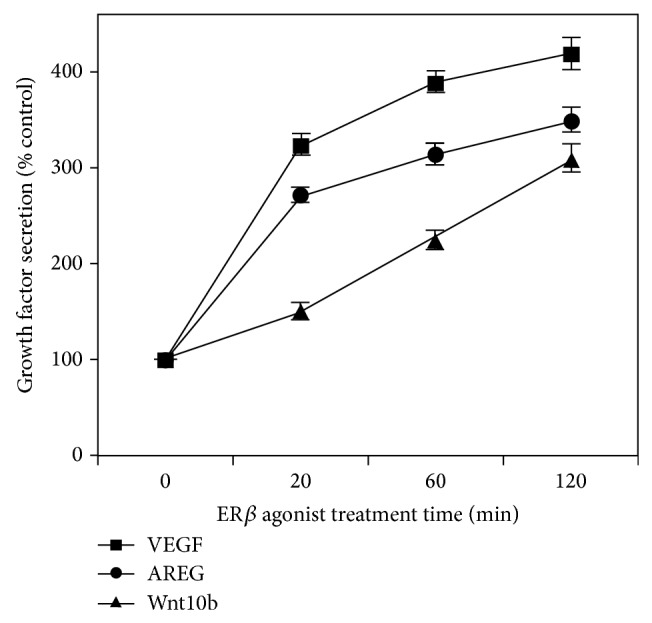
ER*β*1 stimulates secretion of VEGF, amphiregulin, and WNT 10b/12. TNBC cells (MDA-MB-231) were cultivated in estrogen-free media and then treated for 20–120 minutes with ER*β* agonist (10 nM DPN), followed by harvesting of medium and ELISA assays for VEGF, amphiregulin (AREG), and WNT 10b/12, signaling molecules that are each reported to contribute to the progression of TNBC [52, 53, 64]. Assays were done in triplicate in 3 independent experiments. DPN promoted secretion of several critical growth factors (all at *P* < 0.001) as compared to appropriate controls.

To investigate potential cross talk between ER*β*1 and growth factor signaling pathways [15] which may promote TNBC progression (such as VEGF, amphiregulin, and WNT 10b/12) [1, 15, 52, 53, 64], we cultured TNBC cells in the presence and absence of DPN. Using established ELISA methods as detailed before [51], levels of selected secreted growth factors were assayed in particle-free, extracellular media (Figure 5). DPN promoted secretion of several critical growth factors (*P* < 0.001).

### 3.5. Activation of ER*β*1 Correlates with an Increase in EGFR Expression and Activation of Downstream Signaling Pathways

**Figure 6:**
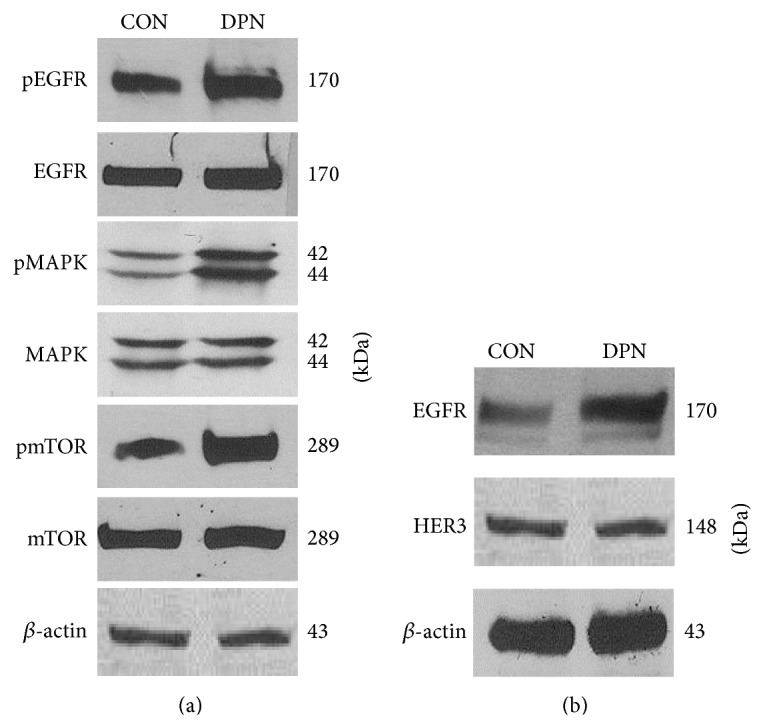
Estrogen receptor-*β* agonist DPN promotes expression and activation of EGFR and downstream signaling in MDA-MB-231 cells. (a) Cells were treated with DPN for 15 minutes. Thereafter, cells were lysed and processed for gel electrophoresis and Western immunoblot using antibodies against phosphotyrosine-1068- and total-EGFR, phospho-p44/42- and total-MAPK, and phosphoserine-2448- and total-mTOR. These data are consistent with independent reports on DPN activity [66]. (b) MDA-MB-231 cells were treated with DPN for 24 hours. Then, cells were lysed, processed for gel electrophoresis and Western immunoblot using antibodies to EGFR and HER-3. *β*-actin was the loading control. Blot shown is representative of at least three independent experiments.

Since EGFR is known to be expressed and active in many TNBCs [1, 64], we explored cross communication between ER*β* activation and EGFR in TNBC cells. MDA-MB-231 cells treated with DPN for 15 minutes showed increased phosphorylation of Tyr1068-EGFR as well as downstream signaling demonstrated by phosphorylation of p44/p42-MAPK and phospho-Ser2448-mTOR (see Figure 6(a)). Further, TNBC cells treated with DPN for 24 hours had increased EGFR protein levels but levels of HER-3, a related EGFR family member, were not increased (Figure 6(b)). Our findings in Figure 5 that note ER*β*-induced promotion of amphiregulin, a known EGFR ligand, as well as EGFR expression (Figure 6(b)) implicate a cascade that could potentially promote downstream EGFR signaling modules such as the Ras/Raf/MEK/ERK1/2 and mTOR pathway for TNBC progression [15, 65]. Hence, ER*β*1 elicits increased phosphorylation of EGFR, as well as activation of MAPK and mTOR.

### 3.6. IGF-2 Stimulates ER*β* Transcription

**Figure 7:**
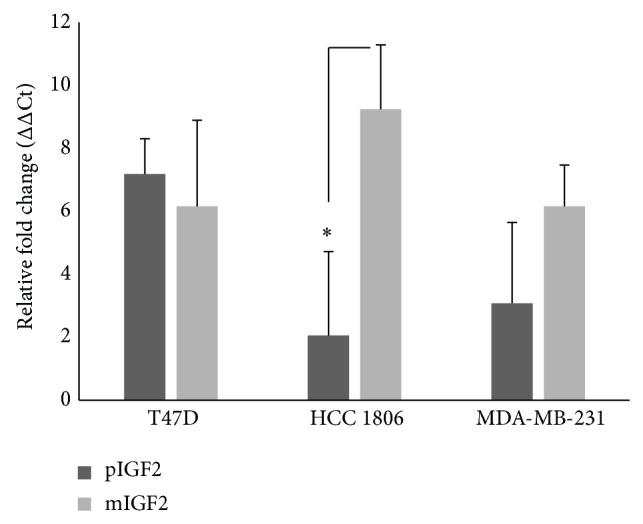
IGF-2 modulates the transcription of ER*β* in HCC 1806 and MDA-MB-231 cell lines. Cells were seeded and then cultured for 24 hours in serum-free, phenol red-free media. Following serum starvation, cultures were treated with 100 ng/mL of pIGF-2 or mIGF-2 (GroPep). Following a 24-hour incubation period, total RNA was extracted and qRT-PCR was performed. Data presented are the result of at least six independent experiments performed in triplicate. Statistical significance was determined using Student's *t*-test (Graphpad). Error bars: SE. ^*^ *P*  value = 0.05.

To evaluate potential effects of unprocessed or big (pIGF2) and processed (mIGF2) IGF-2 on ER*β* transcription, we used a real-time PCR approach. As compared to T47D cells, which express both ER*α* and ER*β*, higher transcript levels of ER*β* were stimulated by mIGF-2 in HCC 1806 and MDA-MB-231 cell lines (Figure 7). These findings indicate a role of IGF-2 in ER*β* transcription.

### 3.7. IGF-2 and ER*β*1 Expression in Archival TNBC Specimens

**Figure 8:**
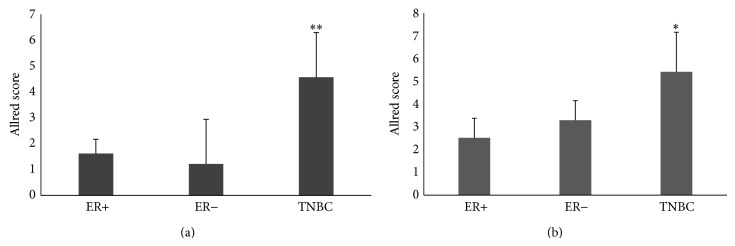
High levels of IGF-2 occur in archival TNBC tumors as compared to ER*α*+ and ER*α*− breast cancers. Archival breast tissues from patients with ER*α*-positive (ER+, *n* = 5), ER*α*-negative (ER−, *n* = 5), and TNBC (*n* = 5) breast cancer were evaluated for IGF-2 expression. Validated IHC staining methods were used to evaluate IGF-2 expression in tumor epithelium (a) and neighboring stroma (b). Scoring was done using an Allred-based criterion. (a) Significantly higher expression of IGF-2 is found in the epithelium of TNBC tumors as compared to ER+ (^**^ *P* < 0.01) and ER− breast cancers (^**^ *P* < 0.01). (b) Compared to ER+ and ER− archival breast tumors, stromal tissue adjacent to TNBC tumors expressed significantly higher levels of IGF-2 than stromal tissue neighboring non-TNBCs (^**^ *P* < 0.01 and ^*^ *P* < 0.05 for ER+ and ER−, resp.). Error bars: SD.

IGF-2 is a secreted protein highly expressed in breast tumor tissue. To assess the association of elevated IGF-2 with a specific tumor subtype, we evaluated IGF-2 expression levels in epithelial and neighboring stromal components of ER*α*-positive (ER+), ER*α*-negative (ER−), and TNBC tumors. Following pathology review, we obtained archival tissue from 5 patients in each subtype. The patients selected ranged in age from 35 to 61 years (average age 50) with additional demographic characteristics shown in Table 3. In comparison to ER+ and ER− epithelium, TNBC epithelia expressed significantly higher levels of IGF-2 (*P* < 0.01; see Figure 8(a)). Additionally, IGF-2 staining in neighboring stromal cells was highest in TNBCs as compared to ER− tumors (*P* = 0.05) and ER+ tumors (*P* < 0.01; see Figure 8(b)). These results support a potential apocrine and/or paracrine role of IGF-2 in cancer progression.

**Figure 9:**
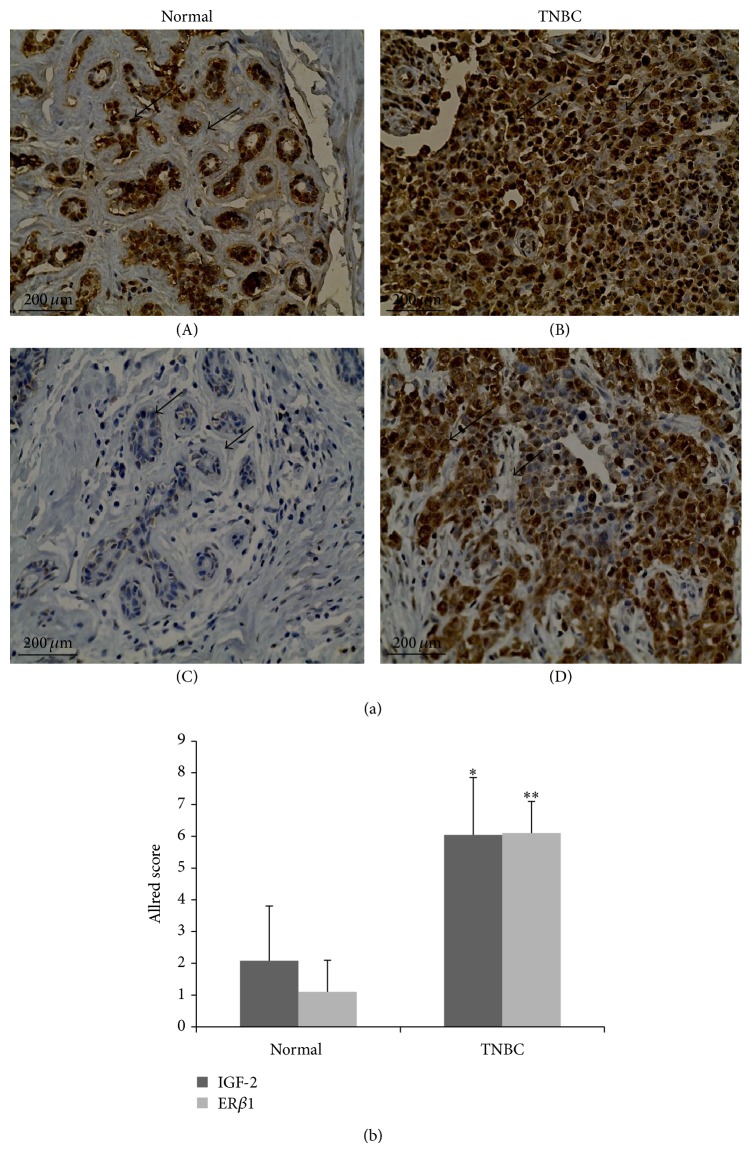
IGF-2 and ER*β*1 are highly expressed in TNBC tumors. Archival breast tissue from patients with TNBC along with adjacent normal tissue adjacent to TNBC tumors was obtained from the NIH CHTN and UCLA Early Detection Research Network. Immunohistological assays were used to evaluate tissue samples for IGF-2 and ER*β*1 expression (see Section 2). (a) Representation of IGF-2 and ER*β*1 expression in normal and TNBC breast tissue. IGF-2 (A and B) and ER*β*1 (C and D) expression were evaluated and visualized using Evos xl Core microscope. Representative images are presented. (b) Allred scoring of IGF-2 and ER*β*1 in archival breast tissue. Samples from neighboring normal tissue (*n* = 7) and from TNBC tumors (*n* = 11) were stained for IGF-2 and ER*β*1 then scored using Allred-based criterion (see Section 2). IGF-2 (^**^ *P* < 0.01) and ER*β*1 (^***^ *P* < 0.005) were notably expressed in TNBC tumor samples. Statistical significance was determined using Student's *t*-test (Graphpad). Error bars: SE.

Since the expression of IGF-2 and ER*β*1 is associated with poor patient outcomes, we evaluated their coexpression in normal breast tissue and TNBC tumors (Figure 9). Using immunohistochemistry, archival TNBC tumors and adjacent nonmalignant breast specimens were evaluated for IGF-2 and ER*β*1 expression (Figure 9(a)). In the samples evaluated and scored, notably increased expression of IGF-2 (*P* < 0.01) and ER*β*1 (*P* < 0.005) is found in all TNBC tumors examined (Figure 9(b)).

## 4. Discussion

The discovery of ER*β* and its expression in TNBC raised hope that targeting ER*β* might offer new treatment options for TNBC patients where previously only aggressive chemotherapies were available [1, 2, 17]. However, these predictions have yet to be realized. Some investigators report that ER*β* is a favorable prognostic factor [13] or tumor suppressor [10], while others find that ER*β* correlates with aggressive phenotypes and worse prognosis [8, 14, 17, 58]. These differences may be due in part to use of nonspecific antibodies to assay ER*β* and some studies of archival specimens lack validation of a true TNBC phenotype [17, 20, 21]. To address these problems, we used validated ER*β* antibodies [58, 67] and established TNBC specimens (ER*α*-negative/PR-negative/HER2-overexpression-negative). Our findings support earlier reports on the prognostic potential of ER*β* isoforms in TNBC [7, 8, 11, 17, 20, 21, 68, 69], particularly ER*β*1. Our data show specific staining for ER*β*1 isoform in TNBC specimens, a finding consistent with other recent reports that both ER*β*1 and ER coregulator SRAP are predictive biomarkers of tamoxifen-response/benefit in women with ER*α*-negative breast cancer [17, 70]. It is independently reported that high levels of nuclear ER*β*2 are associated with lymph node involvement and serve as an independent biomarker for early tumor relapse in ER*α*-negative breast cancers, particularly in TNBC subgroups [69]. Since ER*β*1 and ER*β*2 tend to correlate positively, ER*β*2 expression may contribute in part to this finding. This may be critical as ER*β*2 can heterodimerize with ER*β*1 and modulate gene expression [22, 68]. It will be important in future work to assess the ratio of ER*β*1 : ER*β*2 expression in a larger sample of TNBC specimens and to correlate receptor protein and transcript levels with treatment outcomes [21]. Expression of ER*β*1 is of particular interest because it is the only ER*β* isoform that contains an intact LBD, thereby serving as a potential drug target in the clinic [17, 21, 22].

In pursuit of new approaches to treat TNBC, it is important to consider results of some recent studies on TNBC and related ER*α*-negative disease. Our data appear consistent with independent reports of ER*β*1 as a biomarker for improved survival in TNBC patients when treated with tamoxifen [13, 17, 21, 70]. Although previous work suggests that tamoxifen use only reduced the risk of ER*α*-positive breast cancer, Yan et al. [17] report that both ER*β*1 and ER coregulator SRAP are predictive biomarkers of tamoxifen-response/benefit in women with ER*α*-negative breast cancer. In ER*α*-negative tumors, ER*β*1 expression correlates with Ki-67 proliferation marker suggesting that ER*β*1 may have a role in driving proliferation; thus antiestrogen treatment aimed at ER*β*1 inhibition could slow tumor progression [17]. With the discovery of ER*β* expression alone in TNBC, this presents the possibility that, in this ER*α*-negative cohort, antiestrogen strategies may potentially mediate activity via ER*β*1, the full-length ligand-binding receptor isoform [57, 71, 72].

Although earlier work confirmed that ER*α*-positive breast cancer cells are more sensitive than ER*α*-negative breast cells to the growth-inhibitory effects of tamoxifen, moderate antiproliferative responses to tamoxifen and to ICI-164,384 are found in ER*α*-negative cells [73–75]. These effects may be modulated in part by a second unique binding site identified for hydroxytamoxifen in the coactivator-binding groove of ER*β* that may disrupt ER*β*-coactivator interactions [76]. Collectively, these reports have important implications since approved breast cancer treatments (tamoxifen, raloxifene) are largely well tolerated and orally administered. Such medications may be alternative treatments for ER*β*-positive TNBC patients with generally few options other than cytotoxic chemotherapy [1, 17, 21]. A clinical trial to test this hypothesis in TNBC patients with ER*β*-positive status was recently launched [77].

We note that ER*β* activity is generally considered antagonistic to that of ER*α* when both receptors occur together in a cell, but, in isolation, the role of ER*β* forms is not well-documented. Molecular studies show that when both ER*α* and ER*β* are present together in tumor cells, each ER restricts the binding site occupancy of the other, with ER*α* generally being dominant to ER*β*. It is clear that ER*β* binding and actions in gene regulation are different in the absence of ER*α* expression in breast tumor cells [78]. Indeed, correlation of ER*β* with high proliferative biomarkers is reported in ER*α*-negative tumors but not in those with ER*α* expression [13, 17]. Among the first studies on stable transfection of ER*β* in MDA-MB-231 cells, it was determined that the proliferation rate of the tumor cells positively correlates with the level of ER*β* expression [23]. Results using stable ER*β* clones in ER*α*-negative tumor cells demonstrate increased proliferation as ER*β* expression increased. A confirmation of these experiments was published when MDA-MB-435 cells transfected with ER*β* led to significant stimulation of tumor progression as well as metastasis* in vivo* [79]. These reports support ER*β* stimulated tumor proliferation in ER*α*-negative breast cancer cells [23, 79] and complement the data presented in this study. In contrast, in other studies with ER*β* transfection in ER*α*-low or -negative breast cancer cells, cell proliferation was inhibited [80, 81]. Several reasons may explain such contrasting results. For example, transfection of ER*α* led to the paradoxical finding that ER*α* was a growth inhibitor in breast cancer, a result that is clearly inconsistent with established clinical findings [82–84]. This unexpected result and similar ER*β* transfection data may be due, in part, to excessive levels of ER expression in the model systems used or other complicating factors [23]. Further, conflicting data could be the result of differences in ER*β*1 and ER*β*2 expression/activity. High nuclear ER*β*2 is an independent marker of early relapse in ER*α*-negative breast cancer and especially TNBC [69]. As noted above, it will be important to assess the impact of ER*β*2 [22]. It is possible that ER*β*1 and ER*β*2 are both necessary partners in promoting aggressive TNBC [85]. Finally, there are conflicting reports on activity of ER*β*-specific ligands in TNBC as well as other tumor types [86, 87]. ER*β*1 expression correlates with tumor proliferation and progression in lung cancer [58, 87–89] but not in colon cancer [90]. An important new report with direct relevance to TNBC shows that under basal conditions ER*β* agonists induced apoptosis in breast cancer cells [65]. However, when extracellular signal-regulated kinases 1 and 2 (ERK1/2) were activated by coincubation with epidermal growth factor (EGF), ER*β*1 agonist DPN induced proliferation in breast cancer cells [65]. Such results indicate EGFR-induced signaling activity, known to be frequently overexpressed and active in TNBCs, can modulate ER*β* growth-promoting effects [15, 91].

Emerging evidence highlights the increased frequency of obese patients afflicted with TNBC [25, 92–94] and a recent meta-analysis indicates that obese women are at a greater risk of presenting with a TNBC than nonobese women [95]. The insulin family of growth factors are notably critical mediators of metabolic factors [25, 93]. IGF-2 expression is strongly enhanced in invasive breast cancers and stimulates downstream mTOR signaling and TNBC cell migration [29, 41]. An IGF gene signature including those involved in cell growth, survival, metabolism, and biosynthesis is activated in TNBC and ER*α*-negative breast tumors [96, 97]. Of note, this IGF signature altered mRNA levels of numerous members of both the PI3K and ERK1/2 pathways with enrichment for transcriptional targets of PI3K/Akt/mTOR and EGFR/Ras/MAPK pathways.

Our findings show that ER*β* transcripts are stimulated severalfold in TNBC cells treated with IGF2 and elevated IGF-2 expression occurs in both archival TNBC and neighboring stromal cells. These data are consistent with independent reports that detected IGF-2 in both tumor stroma and epithelial breast cancer cells and correlated IGF-2 expression with breast epithelial [27, 43–45] and stromal cell proliferation [46]. Westley and May [26] also report cross communication between estrogen and IGF pathways, with estrogen promoting increased production of IGF-2. Extensive cross talk between the IGF signaling pathway and two major growth regulators in breast cancer, namely, ER and EGFR, is detailed in numerous studies [15, 33]. Previous ideas about why patients with TNBC have more aggressive tumors that relapse earlier than patients with other types of breast cancer have focused only on tumor cell-intrinsic properties. Our results support the notion that the host systemic environment and local stromal cell niches may also play a role in tumor behavior [98]. Cross talk between epithelium and stroma is essential for neoplastic transformation in many hormonally regulated tissues [99, 100]. One way this cross talk occurs is through reciprocal signals regulating hormone receptor expression or activity in each cellular compartment. In patients with TNBC, progression of tumor cell populations may depend in part on complex interactions and the bioavailability of IGF-2 and ER*β* [98].

## 5. Conclusions

Results of this study indicate that ER*β*1 expression in archival TNBC specimens associates with significantly worse 5-year overall survival, a common characteristic of this disease. Significant expression of ER*β* in a panel of TNBC cell lines is consistent with these findings. Of note, IGF-2, known to associate with breast cancer promotion, is expressed in TNBC and neighboring cells in archival clinical specimens and stimulates increased levels of ER*β* mRNA in TNBC cells. Using shRNA to silence ER*β* expression, we find that ER*β* suppression significantly reduced TNBC proliferation. ER*β*-specific antagonists similarly reduced TNBC growth. In TNBC, ER*β* may stimulate growth through downstream actions that promote VEGF, amphiregulin, and Wnt-10b secretion which in turn activate specific receptor signaling pathways known to be associated with TNBC progression. This work may further our understanding on the interplay of metabolic and growth factors in TNBC, and, hopefully, lead to new therapeutics to manage patients afflicted with this deadly disease.

## Figures and Tables

**Table 1:** Demographic characteristics.

Variable	*N* (%)
Race	
African American	3 (21.4)
Caucasian	11 (78.6)
AJCC stage	
0-I	0
II	7 (50)
III-IV	7 (50)
Ki-67 status	
Low	3 (21.4)
High	11 (78.6)
Age, mean years (range)	
<50 yrs	8 (32–58)
>50 yrs	6 (53–65)

**Table 2:** ER*β*1 biomarker distribution.

Variable	*N* (%)
Nuclear ER*β*1 primary tumor	
Positive	8 (57.1)
Negative	6 (42.8)

**Table 3:** Demographic characteristic of archival ER+, ER−, and TNBC breast samples.

Variable	ER+	ER−	TNBC
Race	**ER*α*+/PR+/HER2+**	**ER*α*−/PR−/HER2+**	**ER−/PR−/HER2−**
African American	3 (60%)	1 (20%)	2 (40%)
Caucasian	2 (40%)	4 (80%)	3 (60%)
AJCC stage			
0-I	2 (40%)	None	1 (20%)
II	2 (40%)	None	None
III-IV	1 (20%)	5 (100%)	4 (80%)
Ki-67 status			
Not recorded	4 (80%)	1 (20%)	1 (20%)
Low	1 (20%)	1 (20%)	1 (20%)
High		3 (60%)	3 (60%)
Metastasis			
Not recorded			1 (20%)
Positive	2 (40%)	3 (60%)	2 (40%)
Negative	3 (60%)	2 (40%)	2 (40%)
Age, mean years (range)			
<50 yrs	3 (45–49)	2 (35–49)	2 (45–49)
>50 yrs	2 (50–70)	3 (50–70)	3 (50–70)

## References

[1] Foulkes W. D., Smith I. E., Reis-Filho J. S. (2010). Triple-negative breast cancer. *The New England Journal of Medicine*.

[2] Morris G. J., Naidu S., Topham A. K. (2007). Differences in breast carcinoma characteristics in newly diagnosed African-American and Caucasian patients: a single-institution compilation compared with the national cancer institute's surveillance, epidemiology, and end results database. *Cancer*.

[3] Amirikia K. C., Mills P., Bush J., Newman L. A. (2011). Higher population-based incidence rates of triple-negative breast cancer among young African-American women. Implications for breast cancer screening recommendations. *Cancer*.

[4] Stark A., Kleer C. G., Martin I. (2010). African ancestry and higher prevalence of triple-negative breast cancer. *Cancer*.

[5] Madak-Erdogan Z., Kieser K. J., Sung H. K., Komm B., Katzenellenbogen J. A., Katzenellenbogen B. S. (2008). Nuclear and extranuclear pathway inputs in the regulation of global gene expression by estrogen receptors. *Molecular Endocrinology*.

[6] (1998). Tamoxifen for early breast cancer: an overview of the randomised trials. Early Breast Cancer Trialists' Collaborative Group. *The Lancet*.

[7] Skliris G. P., Leygue E., Curtis-Snell L., Watson P. H., Murphy L. C. (2006). Expression of oestrogen receptor-*β* in oestrogen receptor-*α* negative human breast tumours. *British Journal of Cancer*.

[8] Novelli F., Milella M., Melucci E. (2008). A divergent role for estrogen receptor-beta in node-positive and node-negative breast cancer classified according to molecular subtypes: an observational prospective study. *Breast Cancer Research*.

[9] Jensen E. V., Cheng G., Palmieri C. (2001). Estrogen receptors and proliferation markers in primary and recurrent breast cancer. *Proceedings of the National Academy of Sciences of the United States of America*.

[10] Hartman J., Strom A., Gustafsson J. A. (2009). Estrogen receptor beta in breast cancer-diagnostic and therapeutic implications. *Steroids*.

[11] Chen J.-Q., Russo J. (2009). ER*α*-negative and triple negative breast cancer: molecular features and potential therapeutic approaches. *Biochimica et Biophysica Acta*.

[12] Phipps A. I., Chlebowski R. T., Prentice R. (2011). Reproductive history and oral contraceptive use in relation to risk of triple-negative breast cancer. *Journal of the National Cancer Institute*.

[13] Honma N., Horii R., Iwase T. (2008). Clinical importance of estrogen receptor-*β* evaluation in breast cancer patients treated with adjuvant tamoxifen therapy. *Journal of Clinical Oncology*.

[14] Poola I., Fuqua S. A. W., de Witty R. L., Abraham J., Marshallack J. J., Liu A. (2005). Estrogen receptor *α*-negative breast cancer tissues express significant levels of estrogen-independent transcription factors, ER*β*1 and ER*β*5: potential molecular targets for chemoprevention. *Clinical Cancer Research*.

[15] Pietras R. J., Márquez-Garbán D. C. (2007). Membrane-associated estrogen receptor signaling pathways in human cancers. *Clinical Cancer Research*.

[16] Hammes S. R., Levin E. R. (2007). Extranuclear steroid receptors: nature and actions. *Endocrine Reviews*.

[17] Yan Y., Li X., Blanchard A. (2013). Expression of both estrogen receptor-beta 1 (ER-*β*1) and its co-regulator steroid receptor RNA activator protein (SRAP) are predictive for benefit from tamoxifen therapy in patients with estrogen receptor-alpha (ER-*α*)-negative early breast cancer (EBC). *Annals of Oncology*.

[18] Wessler S., Otto C., Wilck N., Stangl V., Fritzemeier K.-H. (2006). Identification of estrogen receptor ligands leading to activation of non-genomic signaling pathways while exhibiting only weak transcriptional activity. *The Journal of Steroid Biochemistry and Molecular Biology*.

[19] Paech K., Webb P., Kuiper G. G. J. M. (1997). Differential ligand activation of estrogen receptors ER*α* and ERr*β* at AP1 sites. *Science*.

[20] Shaaban A. M., Green A. R., Karthik S. (2008). Nuclear and cytoplasmic expression of ER*β*1, ER*β*2, and ER*β*5 identifies distinct prognostic outcome for breast cancer patients. *Clinical Cancer Research*.

[21] Murphy L. C., Leygue E. (2012). The role of estrogen receptor-*β* in breast cancer. *Seminars in Reproductive Medicine*.

[22] Leung Y. K., Mak P., Hassan S., Ho S. M. (2006). Estrogen receptor (ER)-beta isoforms: a key to understanding ER-beta signaling. *Proceedings of the National Academy of Sciences of the United States of America*.

[23] Tonetti D. A., Rubenstein R., DeLeon M. (2003). Stable transfection of an estrogen receptor beta cDNA isoform into MDA-MB-231 breast cancer cells. *Journal of Steroid Biochemistry and Molecular Biology*.

[24] Shanle E. K., Zhao Z., Hawse J. (2013). Research resource: global identification of estrogen receptor *β* target genes in triple negative breast cancer cells. *Molecular Endocrinology*.

[25] Belardi V., Gallagher E. J., Novosyadlyy R., Leroith D. (2013). Insulin and IGFs in obesity-related breast cancer. *Journal of Mammary Gland Biology and Neoplasia*.

[26] Westley B. R., May F. E. B. (1994). Role of insulin-like growth factors in steroid modulated proliferation. *The Journal of Steroid Biochemistry and Molecular Biology*.

[27] Osborne C. K., Clemmons D. R., Arteaga C. L. (1990). Regulation of breast cancer growth by insulin-like growth factors. *Journal of Steroid Biochemistry and Molecular Biology*.

[28] Richardson A. E., Hamilton N., Davis W., Brito C., de León D. (2011). Insulin-like growth factor-2 (IGF-2) activates estrogen receptor-*α* and-*β* via the IGF-1 and the insulin receptors in breast cancer cells. *Growth Factors*.

[29] Mancini M., Gariboldi M. B., Taiana E. (2014). Co-targeting the IGF system and HIF-1 inhibits migration and invasion by (triple-negative) breast cancer cells. *British Journal of Cancer*.

[30] LeRoith D., Baserga R., Helman L., Roberts C. T. (1995). Insulin-like growth factors and cancer. *Annals of Internal Medicine*.

[31] Yang X.-F., Beamer W. G., Huynh H., Pollak M. (1996). Reduced growth of human breast cancer xenografts in hosts homozygous for the lit mutation. *Cancer Research*.

[32] Sciacca L., Costantino A., Pandini G. (1999). Insulin receptor activation by IGF-II in breast cancers: evidence for a new autocrine/paracrine mechanism. *Oncogene*.

[33] Yee D., Lee A. V. (2000). Crosstalk between the insulin-like growth factors and estrogens in breast cancer. *Journal of Mammary Gland Biology and Neoplasia*.

[34] Klotz D. M., Hewitt S. C., Ciana P. (2002). Requirement of estrogen receptor-*α* in insulin-like growth factor-1 (IGF-1)-induced uterine responses and in vivo evidence for IGF-1/estrogen receptor cross-talk. *Journal of Biological Chemistry*.

[35] Vyas S., Asmerom Y., De León D. D. (2005). Resveratrol regulates insulin-like growth factor-II in breast cancer cells. *Endocrinology*.

[36] Singh S. K., Moretta D., Almaguel F., Wall N. R., de León M., de León D. (2007). Differential effect of proIGF-II and IGF-II on resveratrol induced cell death by regulating survivin cellular localization and mitochondrial depolarization in breast cancer cells. *Growth Factors*.

[37] Singh S. K., Moretta D., Almaguel F., de León M., de León D. D. (2008). Precursor Igf-II (proIGF-II) and mature IGF-II (mIGF-II) induce Bcl-2 and Bcl-X_L_ expression through different signaling pathways in breast cancer cells. *Growth Factors*.

[38] Singer C. F., Mogg M., Koestler W. (2004). Insulin-like growth factor (IGF)-I and IGF-II serum concentrations in patients with benign and malignant breast lesions: free IGF-II is correlated with breast cancer size. *Clinical Cancer Research*.

[39] Pacher M., Seewald M. J., Mikula M. (2007). Impact of constitutive IGF1/IGF2 stimulation on the transcriptional program of human breast cancer cells. *Carcinogenesis*.

[40] Livingstone C. (2013). IGF2 and cancer. *Endocrine-Related Cancer*.

[41] Gebeshuber C. A., Martinez J. (2013). MiR-100 suppresses IGF2 and inhibits breast tumorigenesis by interfering with proliferation and survival signaling. *Oncogene*.

[42] Kalla Singh S., Tan Q. W., Brito C., De León M., De León D. (2010). Insulin-like growth factors I and II receptors in the breast cancer survival disparity among African-American women. *Growth Hormone and IGF Research*.

[43] Mathieu M., Rochefort H., Barenton B., Prebois C., Vignon F. (1990). Interactions of cathepsin-D and insulin-like growth factor-II (IGF-II) on the IGF-II/mannose-6-phosphate receptor in human breast cancer cells and possible consequences on mitogenic activity of IGF-II. *Molecular Endocrinology*.

[44] Cullen K. J., Allison A., Martire I., Ellis M., Singer C. (1992). Insulin-like growth factor expression in breast cancer epithelium and stroma. *Breast Cancer Research and Treatment*.

[45] de Leon D. D., Wilson D. M., Powers M., Rosenfeld R. G. (1992). Effects of insulin-like growth factors (IGFs) and IGF receptor antibodies on the proliferation of human breast cancer cells. *Growth Factors*.

[46] Giani C., Campani D., Rasmussen A. (2002). Insulin-like growth factor II (IGF-II) immunohistochemistry in breast cancer: relationship with the most important morphological and biochemical prognostic parameters. *The International Journal of Biological Markers*.

[47] Neve R. M., Chin K., Fridlyand J. (2006). A collection of breast cancer cell lines for the study of functionally distinct cancer subtypes. *Cancer Cell*.

[48] Lehmann B. D., Bauer J. A., Chen X. (2011). Identification of human triple-negative breast cancer subtypes and preclinical models for selection of targeted therapies. *Journal of Clinical Investigation*.

[49] Harrington W. R., Sheng S., Barnett D. H., Petz L. N., Katzenellenbogen J. A., Katzenellenbogen B. S. (2003). Activities of estrogen receptor alpha- and beta-selective ligands at diverse estrogen responsive gene sites mediating transactivation or transrepression. *Molecular and Cellular Endocrinology*.

[50] Meyers M. J., Sun J., Carlson K. E., Marriner G. A., Katzenellenbogen B. S., Katzenellenbogen J. A. (2001). Estrogen receptor-beta potency-selective ligands: structure-activity relationship studies of diarylpropionitriles and their acetylene and polar analogues. *Journal of Medicinal Chemistry*.

[51] Aguilar Z., Akita R. W., Finn R. S. (1999). Biologic effects of heregulin/neu differentiation factor on normal and malignant human breast and ovarian epithelial cells. *Oncogene*.

[52] Wend P., Runke S., Wend K. (2013). WNT10B/*β*-catenin signalling induces HMGA2 and proliferation in metastatic triple-negative breast cancer. *EMBO Molecular Medicine*.

[53] Walsh S., Flanagan L., Quinn C. (2012). mTOR in breast cancer: differential expression in triple-negative and non-triple-negative tumors. *Breast*.

[54] Mandusic V., Dimitrijevic B., Nikolic-Vukosavljevic D., Neskovic-Konstantinovic Z., Kanjer K., Hamann U. (2012). Different associations of estrogen receptor *β* isoforms, ER*β*1 and ER*β*2, expression levels with tumor size and survival in early- and late-onset breast cancer. *Cancer Letters*.

[55] Yoon N. K., Maresh E. L., Elshimali Y. (2010). Elevated MED28 expression predicts poor outcome in women with breast cancer. *BMC Cancer*.

[56] Yoon N. K., Maresh E. L., Shen D. (2010). Higher levels of GATA3 predict better survival in women with breast cancer. *Human Pathology*.

[57] Wu Y., Sarkissyan M., Elshimali Y., Vadgama J. V. (2013). Triple negative breast tumors in African-American and Hispanic/Latina women are high in CD44+, low in CD24+, and have loss of PTEN. *PLoS ONE*.

[58] Mah V., Marquez D., Alavi M. (2011). Expression levels of estrogen receptor beta in conjunction with aromatase predict survival in non-small cell lung cancer. *Lung Cancer*.

[59] Liu X., Minin V., Huang Y., Seligson D. B., Horvath S. (2004). Statistical methods for analyzing tissue microarray data. *Journal of Biopharmaceutical Statistics*.

[60] Márquez-Garbán D. C., Chen H.-W., Fishbein M. C., Goodglick L., Pietras R. J. (2007). Estrogen receptor signaling pathways in human non-small cell lung cancer. *Steroids*.

[61] Weinberg O. K., Marquez-Garban D. C., Fishbein M. C. (2005). Aromatase inhibitors in human lung cancer therapy. *Cancer Research*.

[62] Harvey J. M., Clark G. M., Osborne C. K., Allred D. C. (1999). Estrogen receptor status by immunohistochemistry is superior to the ligand-binding assay for predicting response to adjuvant endocrine therapy in breast cancer. *Journal of Clinical Oncology*.

[63] Janzen D. M., Rosales M. A., Paik D. Y. (2013). Progesterone receptor signaling in the microenvironment of endometrial cancer influences its response to hormonal therapy. *Cancer Research*.

[64] Bilir B., Kucuk O., Moreno C. S. (2013). Wnt signaling blockage inhibits cell proliferation and migration, and induces apoptosis in triple-negative breast cancer cells. *Journal of Translational Medicine*.

[65] Cotrim C. Z., Fabris V., Doria M. L. (2013). Estrogen receptor beta growth-inhibitory effects are repressed through activation of MAPK and PI3K signalling in mammary epithelial and breast cancer cells. *Oncogene*.

[66] Klinge C. M., Blankenship K. A., Risinger K. E. (2005). Resveratrol and estradiol rapidly activate MAP kinase signaling through estrogen receptors *α* and *β* in endothelial cells. *The Journal of Biological Chemistry*.

[67] Wu X., Subramaniam M., Negron V. (2012). Development, characterization, and applications of a novel estrogen receptor beta monoclonal antibody. *Journal of Cellular Biochemistry*.

[68] Leung Y. K., Lam H. M., Wu S. (2010). Estrogen receptor beta2 and beta5 are associated with poor prognosis in prostate cancer, and promote cancer cell migration and invasion. *Endocrine-Related Cancer*.

[69] Chantzi N. I., Tiniakos D. G., Palaiologou M. (2013). Estrogen receptor beta 2 is associated with poor prognosis in estrogen receptor alpha-negative breast carcinoma. *Journal of Cancer Research and Clinical Oncology*.

[70] Mann S., Laucirica R., Carlson N. (2001). Estrogen receptor beta expression in invasive breast cancer. *Human Pathology*.

[71] Phillips K.-A., Milne R. L., Rookus M. A. (2013). Tamoxifen and risk of contralateral breast cancer for *BRCA1* and *BRCA2* mutation carriers. *Journal of Clinical Oncology*.

[72] Litwiniuk M. M., Roznowski K., Filas V. (2008). Expression of estrogen receptor beta in the breast carcinoma of BRCA1 mutation carriers. *BMC Cancer*.

[73] Stewart J., King R., Hayward J., Rubens R. (1982). Estrogen and progesterone receptors: correlation of response rates, site and timing of receptor analysis. *Breast Cancer Research and Treatment*.

[74] von Maillot K., Gentsch H. H., Gunselmann W. (1980). Steroid receptors and response to endocrine treatment and chemotherapy of advanced breast cancer. *Journal of Cancer Research and Clinical Oncology*.

[75] Reddel R. R., Murphy L. C., Hall R. E., Sutherland R. L. (1985). Differential sensitivity of human breast cancer cell lines to the growth-inhibitory effects of tamoxifen. *Cancer Research*.

[76] Wang Y., Chirgadze N. Y., Briggs S. L., Khan S., Jensen E. V., Burris T. P. (2006). A second binding site for hydroxytamoxifen within the coactivator-binding groove of estrogen receptor *β*. *Proceedings of the National Academy of Sciences of the United States of America*.

[77] Kiely B. E., Phillips K. A., Francis P. A. (2011). ANZ1001 SORBET: study of oestrogen receptor beta and efficacy of tamoxifen, a single arm, phase II study of the efficacy of tamoxifen in triple-negative but estrogen receptor beta-positive metastatic breast cancer. *Journal of Clinical Oncology*.

[78] Charn T. H., Liu E. T.-B., Chang E. C., Lee Y. K., Katzenellenbogen J. A., Katzenellenbogen B. S. (2010). Genome-wide dynamics of chromatin binding of estrogen receptors alpha and beta: mutual restriction and competitive site selection. *Molecular Endocrinology*.

[79] Hou Y. F., Yuan S. T., Li H. C. (2004). ER*β* exerts multiple stimulative effects on human breast carcinoma cells. *Oncogene*.

[80] Thomas C., Rajapaksa G., Nikolos F. (2012). ER*β*1 represses basal-like breast cancer epithelial to mesenchymal transition by destabilizing EGFR. *Breast Cancer Research*.

[81] Shanle E. K., Zhao Z., Hawse J. (2013). Research resource: global identification of estrogen receptor *β* target genes in triple negative breast cancer cells. *Molecular Endocrinology*.

[82] Jeng M.-H., Jiang S.-Y., Jordan V. C. (1994). Paradoxical regulation of estrogen-dependent growth factor gene expression in estrogen receptor (ER)-negative human breast cancer cells stably expressing ER. *Cancer Letters*.

[83] Jiang S.-Y., Jordan V. C. (1992). Growth regulation of estrogen receptor-negative breast cancer cells transfected with complementary DNAs for estrogen receptor. *Journal of the National Cancer Institute*.

[84] Lazennec G., Katzenellenbogen B. S. (1999). Expression of human estrogen receptor using an efficient adenoviral gene delivery system is able to restore hormone-dependent features to estrogen receptor-negative breast carcinoma cells. *Molecular and Cellular Endocrinology*.

[85] Navaratnam S., Skliris G., Qing G. (2012). Differential role of estrogen receptor beta in early versus metastatic non-small cell lung cancer. *Hormones and Cancer*.

[86] Nair H. B., Kirma N. B., Ganapathy M., Vadlamudi R. K., Tekmal R. R. (2011). Estrogen receptor-*β* activation in combination with letrozole blocks the growth of breast cancer tumors resistant to letrozole therapy. *Steroids*.

[87] Hershberger P. A., Stabile L. P., Kanterewicz B. (2009). Estrogen receptor beta (ER*β*) subtype-specific ligands increase transcription, p44/p42 mitogen activated protein kinase (MAPK) activation and growth in human non-small cell lung cancer cells. *The Journal of Steroid Biochemistry and Molecular Biology*.

[88] Mah V., Seligson D. B., Li A. (2007). Aromatase expression predicts survival in women with early-stage non-small cell lung cancer. *Cancer Research*.

[89] Stabile L. P., Davis A. L., Gubish C. T. (2002). Human non-small cell lung tumors and cells derived from normal lung express both estrogen receptor alpha and beta and show biological responses to estrogen. *Cancer Research*.

[90] Rudolph A., Toth C., Hoffmeister M. (2012). Expression of oestrogen receptor *Β* and prognosis of colorectal cancer. *British Journal of Cancer*.

[91] Corkery B., Crown J., Clynes M., O'Donovan N. (2009). Epidermal growth factor receptor as a potential therapeutic target in triple-negative breast cancer. *Annals of Oncology*.

[92] Davis A. A., Kaklamani V. G. (2012). Metabolic syndrome and triple-negative breast cancer: a new paradigm. *International Journal of Breast Cancer*.

[93] Jain R., Strickler H. D., Fine E., Sparano J. A. (2013). Clinical studies examining the impact of obesity on breast cancer risk and prognosis. *Journal of Mammary Gland Biology and Neoplasia*.

[94] Sturtz L. A., Melley J., Mamula K., Shriver C. D., Ellsworth R. E. (2014). Outcome disparities in African American women with triple negative breast cancer: a comparison of epidemiological and molecular factors between African American and Caucasian women with triple negative breast cancer. *BMC Cancer*.

[95] Pierobon M., Frankenfeld C. L. (2013). Obesity as a risk factor for triple-negative breast cancers: a systematic review and meta-analysis. *Breast Cancer Research and Treatment*.

[96] Creighton C. J., Casa A., Lazard Z. (2008). Insulin-like growth factor-I activates gene transcription programs strongly associated with poor breast cancer prognosis. *Journal of Clinical Oncology*.

[97] Litzenburger B. C., Creighton C. J., Tsimelzon A. (2011). High IGF-IR activity in triple-negative breast cancer cell lines and tumorgrafts correlates with sensitivity to anti-IGF-IR therapy. *Clinical Cancer Research*.

[98] Castaño Z., Marsh T., Tadipatri R. (2013). Stromal EGF and IGF-I together modulate plasticity of disseminated triple-negative breast tumors. *Cancer Discovery*.

[99] Kuperwasser C., Chavarria T., Wu M. (2004). Reconstruction of functionally normal and malignant human breast tissues in mice. *Proceedings of the National Academy of Sciences of the United States of America*.

[100] Janzen D. M., Rosales M. A., Paik D. Y. (2013). Progesterone receptor signaling in the microenvironment of endometrial cancer influences its response to hormonal therapy. *Cancer Research*.

