# Correction to “Biologic Roles of Estrogen Receptor‐*β* and Insulin‐Like Growth Factor‐2 in Triple‐Negative Breast Cancer”

**DOI:** 10.1155/bmri/9818510

**Published:** 2026-04-06

N. Hamilton, D. Márquez‐Garbán, V. Mah, G. Fernando, Y. Elshimali, H. Garbán, D. Elashoff, J. Vadgama, L. Goodglick, R. Pietras, “Biologic Roles of Estrogen Receptor‐*β* and Insulin‐Like Growth Factor‐2 in Triple‐Negative Breast Cancer,” *BioMed Research International*, 2015, 925703, https://doi.org/10.1155/2015/925703

In the article, there is an error in Figure 6, in which the HER3 blots in Figure 6b were duplicated with the *β*‐actin blots shown in Figure 6a. The correct Figure 6 is shown below.

Figure 6Estrogen receptor‐*β* agonist DPN promotes expression and activation of EGFR and downstream signaling in MDA‐MB‐231 cells. (a) Cells were treated with DPN for 15 min. Thereafter, cells were lysed and processed for gel electrophoresis and Western immunoblot using antibodies against phosphotyrosine‐1068‐ and total‐EGFR, phospho‐p44/42‐ and total‐MAPK, and phosphoserine‐2448‐ and total‐mTOR. These data are consistent with independent reports on DPN activity [66]. (b) MDA‐MB‐231 cells were treated with DPN for 24 h. Then, cells were lysed, processed for gel electrophoresis, and Western immunoblot using antibodies to EGFR and HER‐3. *β*‐Actin was the loading control. The blot shown is representative of at least three independent experiments.

**Figure:**
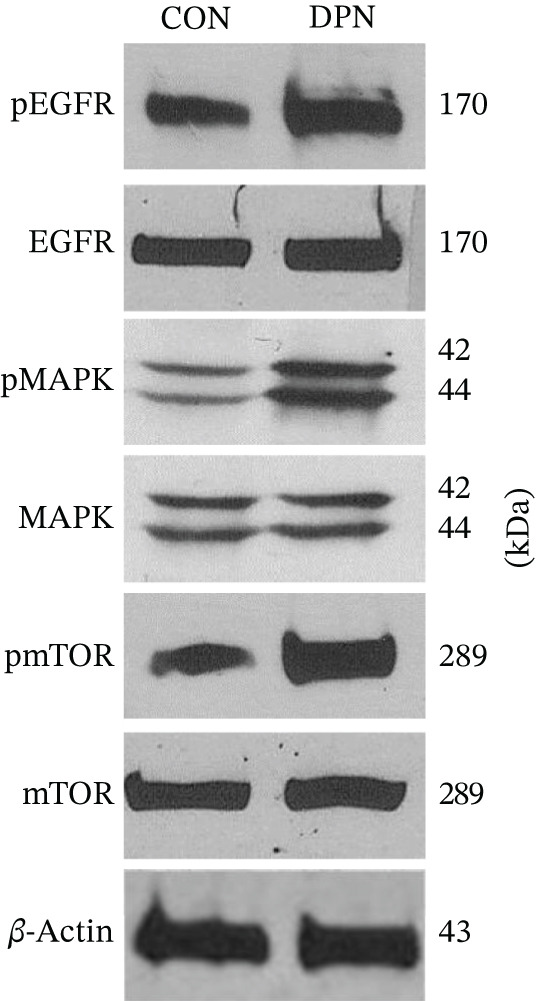
(a)

**Figure:**
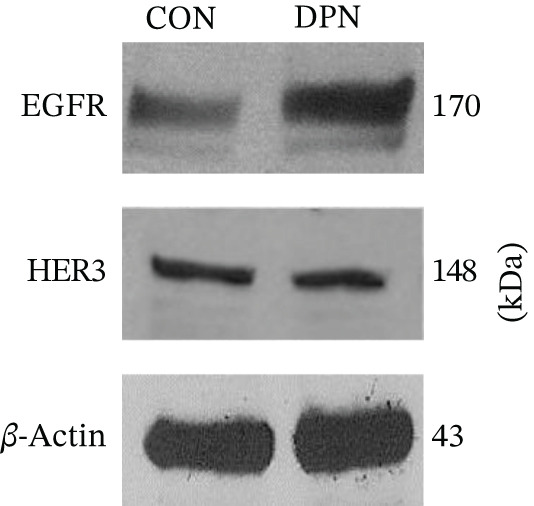
(b)

We apologize for this error.

